# Supplementary material for: Spatiotemporal characters and influence factors of hand, foot and mouth epidemic in Xinjiang, China
Source: PLoS One. 2021 Aug 24;16(8):e0254223. doi: 10.1371/journal.pone.0254223 (PMC8384200; doi:10.1371/journal.pone.0254223)
Supplement: Attachment — Submitted filename: Response to Reviewers.pdf [file pone.0254223.s002.pdf]

Responses to Reviewers

We are very grateful to the reviewers for the very helpful suggestions and critical comments on our revised manuscript. The funding statement, ethics statement, data availability statement and original map statement have been provided in the revised cover letter. According to reviewers' comments, a detailed point-to-point response is provided as follows.

Reviewer 1:

Comment 1. What's the main purpose to show the status of cases. It mainly reflects the distribution of age. It is a well-known knowledge that young children are the main target of HFM disease. May be the specific environmental condition of children staying at home or school in the study area could be discussed.

Response: Thank your suggestion. We have deleted the status of cases in the revision.

Comment 2. Lines 213-215. The statement is confusing. Why do you ignore Kashgar, Kizilsu, and Khotan?

Response: In the revision, we have added the explanation of these three regions (see page 11 lines 220-221).

Comment 3. Lines 231-233. What is the current strategy to block HFM transmission in the study area? Why increasing the frequency of children's health examination can help to reduce transmission?

Response: We have provided the strategy and the purpose of increasing children's health examination in the revision (see page 12 lines 239-244).

Comment 4. Lines 273-281. The climate condition can impact virus survival and transmission. How about socioeconomic factors? The statement only described positive or negative effects of these factors. How can you explain the associations?

Response: Thank your suggestion. We have added the relationship between the six socioeconomic factors and HFM disease in the revision (see pages 12-13 lines 278-290).

Comment 5. Line 16. The model you listed here are usually referred to "statistical models".

Response: Yes, you are right. We have corrected it in the revision (see page 2 line 21).

Comment 6. Figure 4. How about the population distribution in the study area? Incidence rate could be used to compare the results.

Response: According to your suggestion, we have replaced the HFM case with the incidence in Figure 4.

Reviewer 2:

Abstract: The authors did not provide specific conclusion regarding the findings. The conclusion is too general and cannot reach to the point of usefulness or next steps. It would be better if the author can emphasize the significant of the findings for public health implication.

Response: Thank your suggestion. We have reorganized the content in Abstract.

Introduction 1: The authors reviewed some articles and presented previous findings properly, however the authors did not provide rationale on the study area and justification to study all factors. Although the authors tried to present aims of the study between line 20-32, the issues did not present research hypothesis at all. Otherwise, this study is looked very alike a reference number 6 but just changed the study setting.

Response: According to your suggestion, we have provided rationale on the study area and some references to reflect the importance of all factors in the revision (see pages 1-2 lines 8-11 and lines 27-32). Moreover, we have illustrated the difference between Reference 6 and our work (see page 2 lines 27-32).

Introduction 2: Also, statement such as section 2, 3, 4 and 5 did not relevant to the way of presentation in the followings part which use heading rather numbering.

Response: Yes, you are right. We have done it in the revision (see page 2 lines 33-38).

Materials and Methods 1: The authors described source of data clearly but for meteorological data, the authors may consider to explain the data collection of the CMDC for example, satellite, meteorological station located at which area to provide clearly understanding how accurate the meteorological data for each area.

Response: Thank your suggestion. We have explained the point in the revision (see pages 2-3 lines 55-59).

Materials and Methods 2: Since the author used spatial analysis, did the author apply spatial weight matrix?

Response: Yes, we have done it (see page 4 lines 92-97).

Materials and Methods 3: Population structure in each area is different or similar which will affect the comparison of HFMD which strongly related with age. The authors should describe spatial context of each area in Xinjiang in particular from south to north, economic, living condition and so on.

Response: We have provided six socioeconomic factors of 15 regions to reflect the economic trend of Xinjiang in the revision (see page 9 lines 187-193).

Result 1: Table 1 will be the most benefit to the public health profession in Xinjiang, if

the authors present specific rate rather than number of cases except variable "status".

Response: According to your suggestion, we have used the incidence in Table 1.

Result 2: Can the downward trend of children attended school affect HFMD trend in the study area?

Response: Yes, it has some impact. The incidence of children aged 0-5 is higher than others, accounting for 77.5-83.6%. For children attending kindergarten, the incidence is about 40.1%.

Result 3: Was a large outbreak happen in the year 2016? Can it affect spatio-temporal analysis?

Response: We have provided the explanation for the 2016 epidemic in the revision (see page 12 lines 239-242). It can't affect the spatio-temporal analysis, since the GTWPR model has just investigated the non-stationarity of spatio-temporal effects.

Result 4: Figure 4, for comparison among study area, the author may consider to apply standardized rate to present.

Response: Yes, we have done it.

Result 5: The distribution of socioeconomic variables should be described.

Response: Thank your suggestion. We have provided the data of socioeconomic variables in the revision (see page 9 lines 187-193).

Result 6: The author described principle component analysis to merge socioeconomic variables but it is not clear how the model selection was conducted. Since the narrative on page 14 line180-184 are not consistent with table 5.

Response: The model selection has been added (see page 10 lines 207-211). Moreover, We have corrected the associations between the narrative and Table 5 in the revision (see page 9 lines 194-196).

Result 7: The inverse association of meteorological data from north and south are interesting, is there any collinearity of the factors? or culture explanation?

Response: Thank your suggestion. We have added the description in the revision (see page 12 lines 271-276).

Discussion: The author did not well explain the findings but rather compare with previous findings. It would be most interesting if the authors could explain the possible reasons why one factor affect differently to each area or even in opposite way.

Response: We have explained some reasons for the findings in the revision (see page 12 lines 271-276, and lines 278-290).
